# YY1 promotes HDAC1 expression and decreases sensitivity of hepatocellular carcinoma cells to HDAC inhibitor

**DOI:** 10.18632/oncotarget.17196

**Published:** 2017-04-18

**Authors:** Sheng Dong, Xiang Ma, Zusen Wang, Bing Han, Hao Zou, Zehua Wu, Yunjin Zang, Likun Zhuang

**Affiliations:** ^1^ Department of Hepatobiliary and Pancreatic Surgery, The Affiliated Hospital of Qingdao University, Qingdao, 266003, China; ^2^ Institute of Transplantation Science, The Affiliated Hospital of Qingdao University, Qingdao, 266003, China

**Keywords:** YY1, HDAC1, HDAC inhibitor, HCC, drug sensitivity

## Abstract

YY1 is a DNA-binding transcription factor and reported to be involved in cancer progression. Histone deacetylase inhibitor (HDACi) could inhibit proliferation and promote apoptosis of Hepatocellular carcinoma (HCC) cells. However, it is unclear about the roles of YY1 in the sensitivity of HCC cells to HDACi. In this study, firstly, we identified two drug-response profiles to HDACi in HCC cell lines, while our results showed that HDAC1 expression was positively correlated with YY1 in HCC cell lines and primary tumor tissues. Secondly, YY1 decreased the sensitivity of HCC cells to HDACi *in vitro* and *in vivo*. Furthermore, we found that YY1 promoted HDAC1 expression by binding to its promoter, while HDAC1 in turn up-regulated the expression of YY1. In conclusion, our results showed that YY1 could reduce the sensitivity of HCC cells to HDACi and might be a potential therapeutic target in HCC.

## INTRODUCTION

Hepatocellular carcinoma (HCC) is one of the most common and malignant tumors in the world. Moreover, the majority of HCC patients are diagnosed at an advanced stage and conventional chemotherapy is usually ineffective. Until now, the mortality rate of HCC is still very high [1]. In the past few years, more and more studies showed that the molecular targeting therapy provided an opportunity for the treatment of patients with cancers including HCC. For example, Sorafenib, a multikinase inhibitor, is one of the molecular targeted drugs in treating patients with advanced HCC [2]. However, the median life expectancy of HCC patients extended by Sorafenib was not long and many patients developed acquired resistance to sorafenib [3]. Therefore, it is necessary to develop a novel molecular targeting therapy for HCC.

Histone deacetylases (HDACs) are an important family of enzymes which deacetylate the amino group of the lysine residues in the histone tails to form a closed chromatin configuration and played important roles in epigenetic regulation of key genes involved in cancer incidence and progression [4]. Currently, there are four major classes of HDACs including the class I HDACs (HDAC1, 2, 3 and 8), class II HDACs (HDAC4, 5, 6, 7, 9 and 10), Class III HDACs (Sirtuins) and class IV HDACs (HDAC11). More and more evidences indicated that HDACs could affect many cancerous behaviors including cell proliferation, apoptosis and invasion in many cancers including HCC [5, 6]. Consequently, HDAC inhibitor (HDACi) has exhibited important antitumor activities *in vitro* and *in vivo*, and has been used in clinical treatments for patients with cancers [7, 8]. For HCC, pan-HDCA inhibitor resminostat could offer a new therapeutic option for patients with advanced HCC [9], which suggested that HDACi might be an important strategy for HCC treatment.

In recent years, many genes were reported to regulate the sensitivity of cancer cells to chemotherapeutic drugs and be important biomarkers for predicting clinical responses to chemotherapeutic drugs including HDACi. For example, HR23B governed the sensitivity of cutaneous T cell lymphoma cells to HDACi and was demonstrated as a biomarker for tumor cell sensitivity to HDACi [10]. Another report showed that RNH1 suppressed HDACi-induced reactive oxygen species (ROS) and contributed to HDACi resistance in gastric cancer cells [11]. Until now, there have been few reports focusing on the genes regulating the sensitivity of HCC cells to HDACi. In the past few years, the transcription factor Yin-Yang 1 (YY1), which is ubiquitously expressed and highly conserved, has been reported to regulate expressions of downstream genes to affect the sensitivity and resistance of cancer cells to chemotherapeutic drugs [12]. YY1 was also demonstrated to interact with HDACs including HDAC1, 2 and 3 [13–15]. Furthermore, YY1 was reported to be significantly up-regulated in HCC tissues [16]. In view of the facts above, YY1 might play an important role in regulating the sensitivity of HCC cells to HDACi.

In this study, we found that YY1 expression exhibited a positive correlation with HDAC1 in cell lines and tumor tissues of HCC. We also investigated the reciprocal regulation between YY1 and HDAC1, and the roles of YY1 in sensitivity of HCC cells to HDACi *in vitro* and *in vivo*. Our findings revealed a novel role of YY1 in predicting drug sensitivity of HCC.

## RESULTS

### HDACi treatment defines two drug-response profiles in HCC cell lines

**Figure 1:**
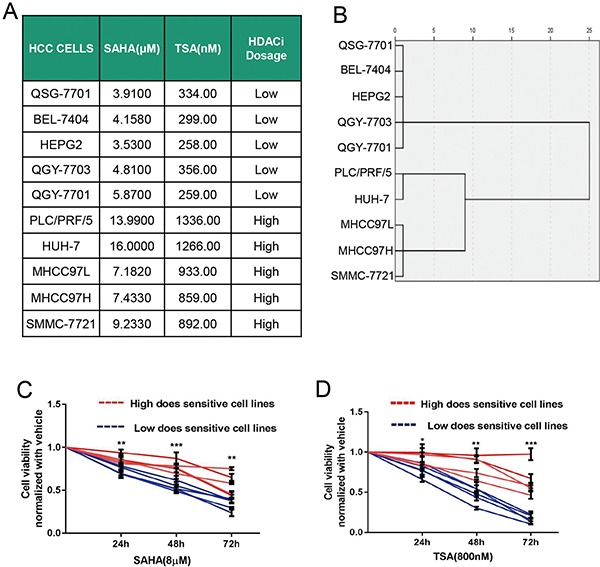
HCC cell lines display two drug-response profiles to HDACi (**A**) IC50 values for 10 HCC cell lines treated with SAHA or TSA at 72 h were determined by CCK-8 assays. (**B**) According to the Cluster analysis with Ward method (Rescaled distance cluster combine), HCC cell lines were classified as sensitive to high or low dose of HDACi. (**C** and **D**) Cell viabilities of different HCC cell lines treated with SAHA (C) or TSA (D) at different time points were measured by CCK8 assays. Five high-dose sensitive HCC cell lines as one group were represented with red curves, while five low-dose sensitive HCC cell lines as another group were represented with blue curves. Cell viability was shown relative to the value obtained in the same cells treated with DMSO under the same condition. Cell viabilities between the two groups were compared with unpaired *t-test* at different time points. *P < 0.05; **P < 0.01; ***P < 0.001.

In order to explore the effects of HDACi on different HCC cell lines, firstly we evaluated the sensitivity of HCC cell lines to non-specific HDACis SAHA and TSA by calculating IC50 values (Figure 1A). According to the ward's cluster analysis, five HCC cell lines were classified as high-dose sensitive cell lines to HDACi with IC50 values above 6 μM for SAHA and 400 nM for TSA, while the other five HCC cell lines were classified as low-dose sensitive cell lines to HDACi (Figure 1A and 1B).

We treated the five high-sensitive cell lines as one group and the five low-sensitive cell lines as another, measured and compared cell viabilities of HCC cells between the two groups treated with SAHA or TSA at different time points. Our results showed that there were more viable cells in high-dose than that in low-dose sensitive HCC cell lines after treatment with SAHA or TSA at various time points (Figure 1C and 1D).

### HDAC1 expression was positively correlated with YY1 in HCC cell lines and tumor tissues

**Figure 2:**
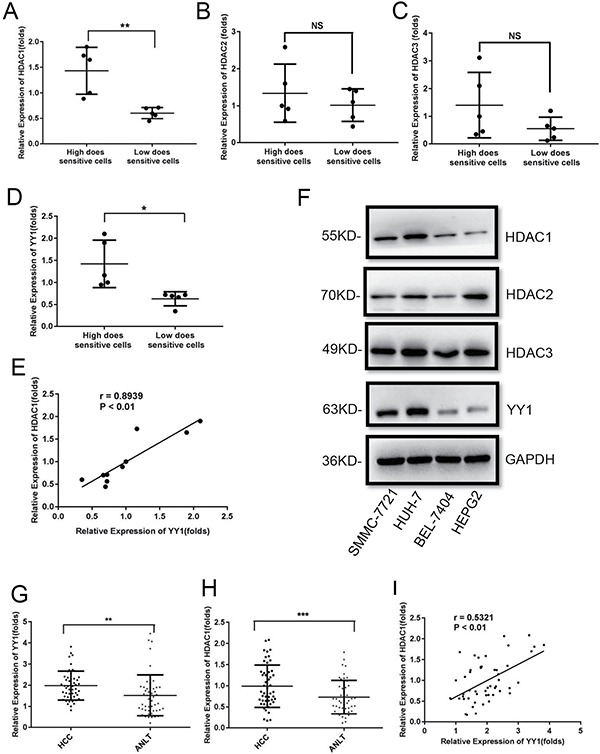
YY1 expression is positively related to HDAC1 in HCC cell lines and tumor tissues (**A**–**D**) The expressions of HDAC1 (A), HDAC2 (B), HDAC3 (C) and YY1 (D) in high and low-dose sensitive HCC cell lines were examined by qRT-PCR. (**E**) The correlation between HDAC1 and YY1 in HCC cell lines. (**F**) Protein levels of HDAC1, 2, 3 and YY1 in different HCC cell lines. (**G** and **H**) Expression levels of YY1 and HDAC1 in 50 pairs of HCC tissues and ANLTs were determined by qRT-PCR. (**I**) The correlation between HDAC1 and YY1 in HCC tissues. *P < 0.05;**P < 0.01; ***P < 0.001; NS, not significant.

To explain the possible mechanism underlying the differential responses of HCC cells to HDACi, firstly, we measured mRNA levels of three main class I HDACs including HDAC1, 2 and 3 in high-dose and low-dose sensitive HCC cell lines. QRT-PCR analysis showed that expression of HDAC1, but not HDAC2 and 3, was significantly higher in high-dose than that in low-dose sensitive HCC cells (Figure 2A–2C). YY1 could be involved in the sensitivity and resistance of cancer cells to chemotherapeutic drugs. Our results also showed that YY1 level was higher in high-dose sensitive HCC cells and exhibited a positive correlation with HDAC1 in HCC cell lines (Figure 2D and 2E). Western blot analysis showed that protein levels of both HDAC1 and YY1 were higher in high-dose sensitive HCC cell lines SMMC-7721 and HUH-7 than that in low-dose sensitive HCC cell lines HEPG2 and BEL-7404 (Figure 2F and Supplementary Figure 1).

Furthermore, we measured HDAC1 and YY1 levels in 50 pairs of HCC tissues and paired adjacent non-tumor tissues (ANLTs). Our results showed that both HDAC1 and YY1 levels were higher in HCC tissues than that in ANLTs (Figure 2G and 2H). Correlation analysis indicated that YY1 levels exhibited a positive relationship with HDAC1 in HCC tissues (Figure 2I).

### YY1 binds to the promoter of HDAC1 and promotes its expression

**Figure 3:**
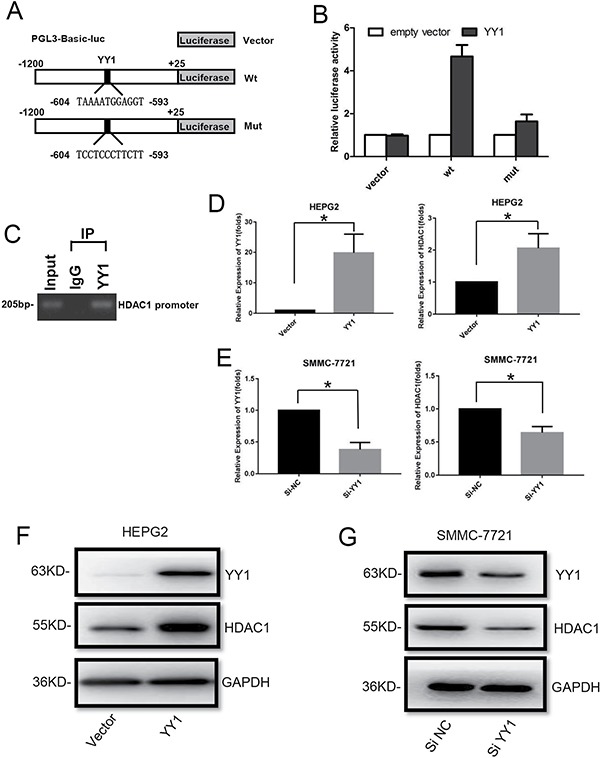
Effect of YY1 on HDAC1 expression (**A**) Schematic diagram of HDAC1 promoter with wide-type (wt) or mutant (mut) YY1 binding sites. (**B**) Luciferase assays were performed in HEPG2 cells transfected with wt or mut promoter. Each luciferase activity was normalized to the value obtained in the cells transfected with vector. (**C**) ChIP assay was used to assess YY1 binding site at the promoter region of HDAC1. (**D**–**G**) QRT-PCR and western blot analysis of YY1 and HDAC1 expression in HEPG2 cells with YY1 overexpression (D and F) and SMMC-7721 cells with YY1 knockdown (E and G). *P < 0.05.

In order to explore the mechanism underlying the correlation between YY1 and HDAC1, we analyzed the promoter of HDAC1 by TransFac and JASPAR database, and one potential binding site of YY1 was identified. Then a 1.2 kb fragment of the human HDAC1 promoter with wide type (wt) or mutant (mut) predicted YY1 binding site was inserted into the luciferase reporter plasmid (Figure 3A). Luciferase reporter analysis showed that overexpression of YY1 led to an increase in luciferase activity of the wt-HDAC1-promoter plasmid in HEPG2 cells, while mut YY1 binding site attenuated the increase of luciferase activity (Figure 3B). In addition, ChIP assay clearly showed that the predicted YY1-binding site in HDAC1 promoter presented the ability to bind to YY1 protein (Figure 3C). Moreover, qRT-PCR and western blot analysis showed that overexpression of YY1 could increase both mRNA and protein levels of HDAC1 in HEPG2 cells (Figure 3D and 3F, Supplementary Figure 2A–2B), while knockdown of YY1 decrease the levels of HDAC1 in SMMC-7721 cells (Figure 3E and 3G, Supplementary Figure 2C–2D).

### YY1 decreases the sensitivity of HCC cells to HDACi *in vitro*

**Figure 4:**
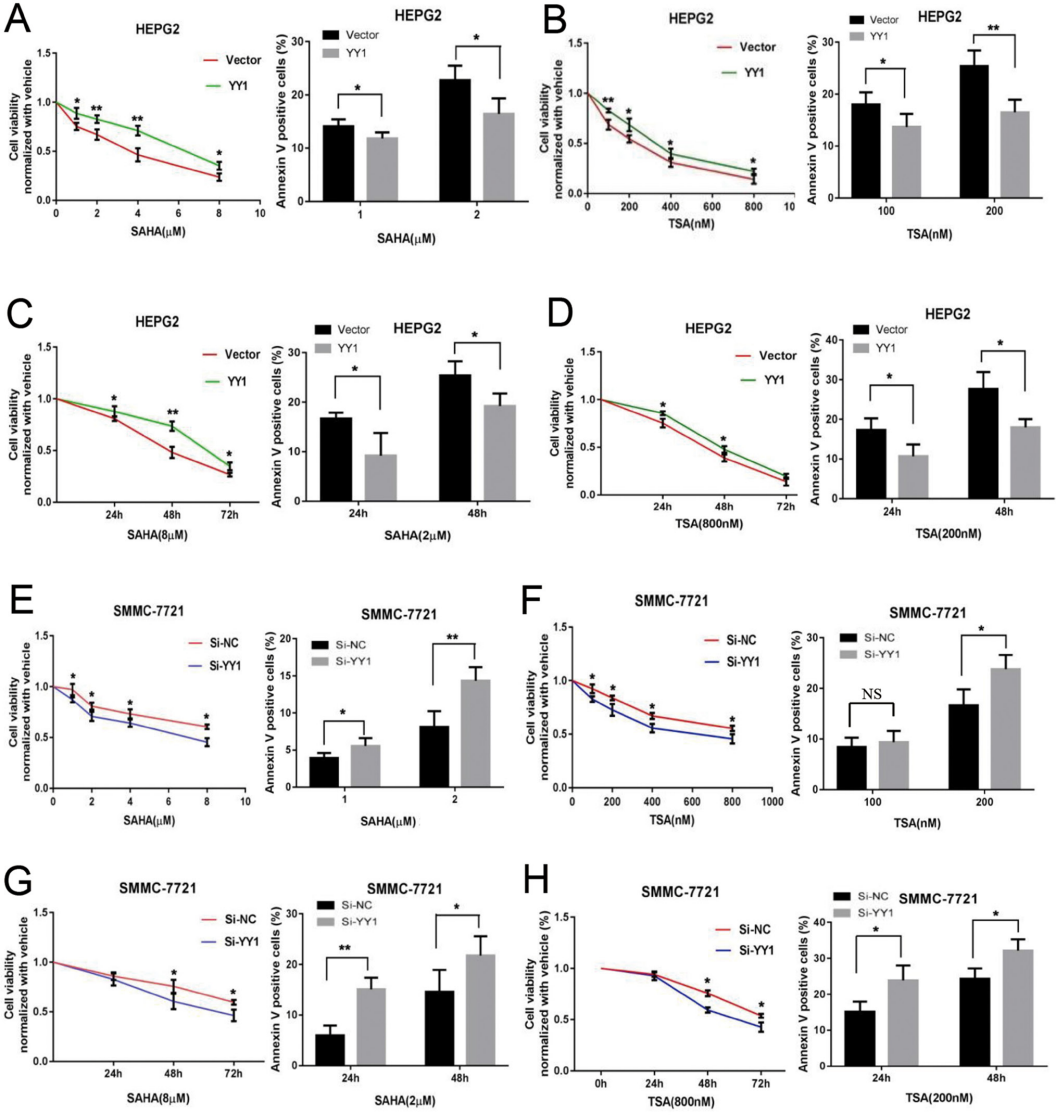
Effects of YY1 on the sensitivity of HCC cells to HDACi *in vitro* SMMC-7721 cells were transfected with YY1-shRNA plasmids and HEPG2 cells were transfected with YY1 expressing plasmids. CCK8 and apoptosis assay were conducted in cells treated with SAHA (**A**, **C**, **E** and **G**) or TSA (**B**, **D**, **F** and **H**) under different drug concentrations at 72 h (A, B, E and F) or under fixed drug concentrations at different time points (C, D, G and H). *P < 0.05; **P < 0.01; NS, not significant.

To determine whether YY1 could affect sensitivity of HCC cells to HDACi, we overexpressed and knocked down YY1 expression in HCC cells, and measured cell proliferation and apoptosis under HDACi treatment at different drug concentrations or time points. The evaluated HDACi concentration and treatment time presented proliferation inhibition and apoptosis induction of HCC cells. Our results showed that YY1 overexpression could significantly promote cell proliferation and inhibit cell apoptosis of HEPG2 cells under HDACi treatments at different drug concentrations and treatment times (Figure 4A–4D). While knockdown of YY1 inhibited proliferation and promoted cell apoptosis of SMMC-7721 cells under HDACi treatments (Figure 4E–4H). These results indicated that YY1 obviously decreased the sensitivity of HCC cells to HDACi *in vitro*.

### Knockdown of YY1 promotes the sensitivity of HCC cells to HDACi *in vivo*

**Figure 5:**
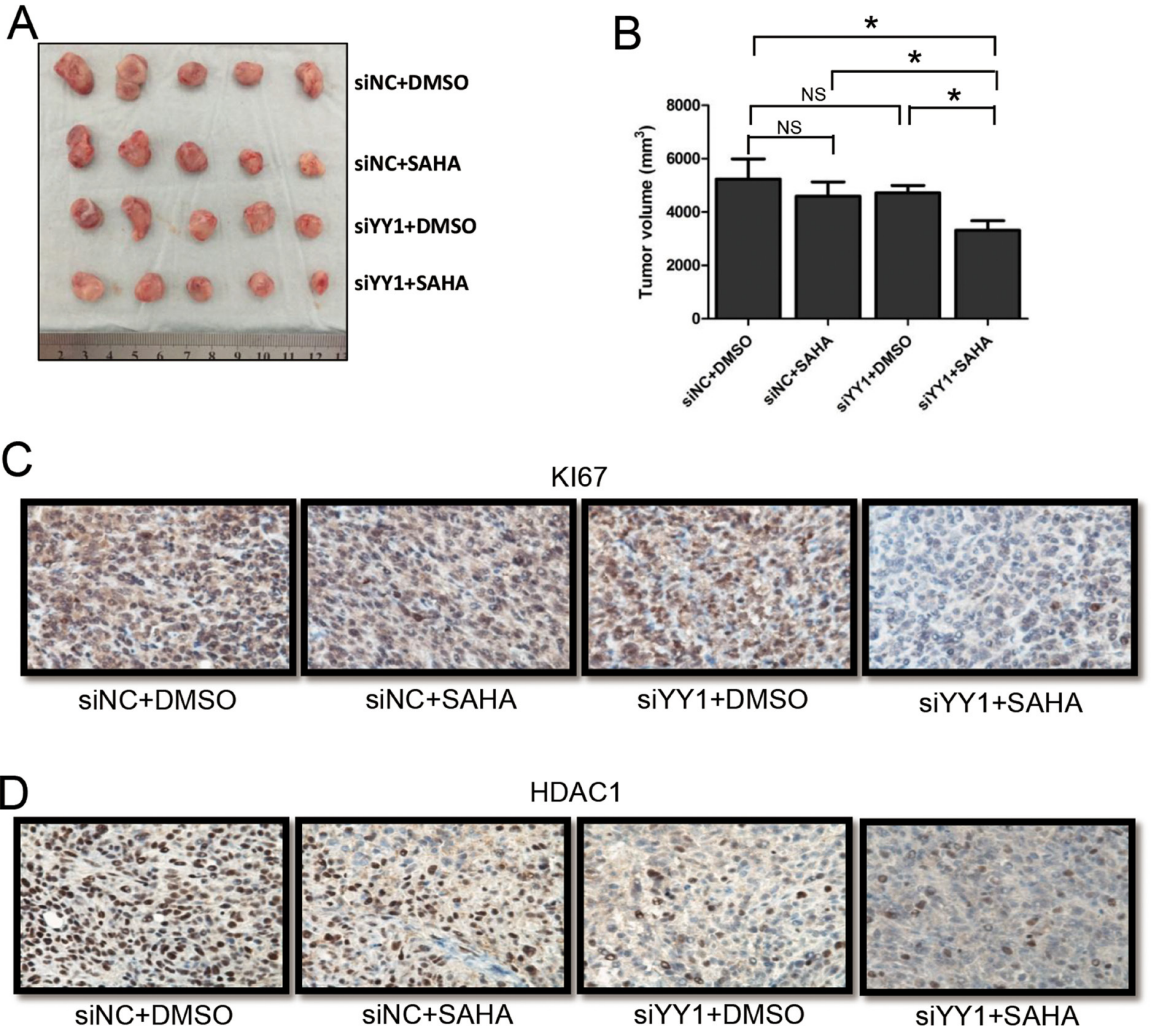
Effects of YY1 on the sensitivity of HCC cells to HDACi *in vivo* (**A**) Morphologic characteristics of tumors from HCC xenograft mice with different treatments were shown. (**B**) Sizes of tumors from mice were calculated and compared in the diagrams. (**C** and **D**) Expressions of KI67 (C) and HDAC1 (D) were examined by immunohistochemistry. *P < 0.05; NS, not significant.

An HCC xenograft mouse model was used to explore the effects of YY1 on HDACi sensitivity of HCC cells *in vivo*. HCC xenograft mice were divided into four groups: siNC plus DMSO, siNC plus SAHA, siYY1 plus DMSO and siYY1 plus SAHA. There was no statistical difference in tumor sizes between siNC plus DMSO group and siNC plus SAHA group. We also found no statistical difference between siNC plus DMSO group and siYY1 plus DMSO group. However, tumor sizes in siYY1 plus SAHA group were significantly smaller than that in any other group (Figure 5A and 5B). Immunohistochemistry assay showed that the expression of proliferation marker KI67 was down-regulated in tumor tissues of siYY1 plus SAHA mouse group (Figure 5C). Accordingly, we found that HDAC1 levels were lower in two siYY1 groups than that in corresponding siNC groups (Figure 5D), suggesting the regulatory effects of YY1 on HDAC1 expression *in vivo*.

### YY1 expression was inhibited by HDACi and up-regulated by HDAC1

**Figure 6:**
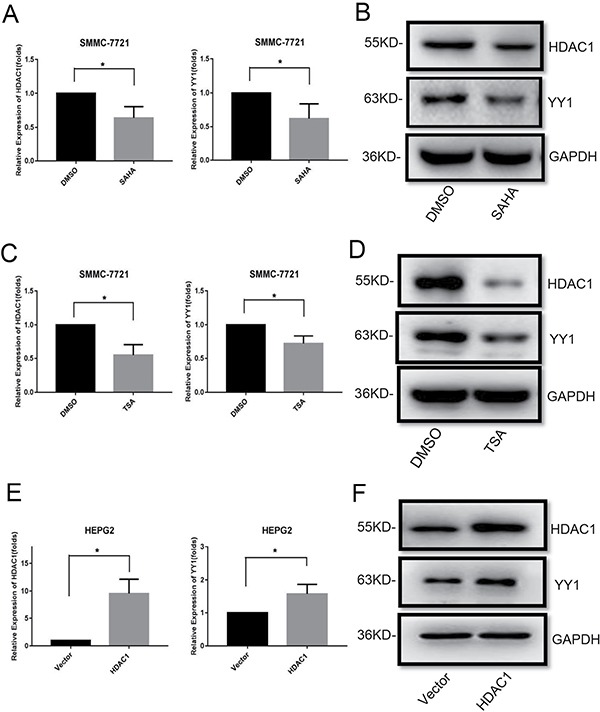
Effects of HDACi and HDAC1 on YY1 expression QRT-PCR and western blot analysis of HDAC1 and YY1 expressions in SMMC-7721 cells treated with HDACis (8 μM SAHA and 800 nM TSA for 48 hours) (**A**–**D**) and HEPG2 cells with HDAC1 overexpression (**E**–**F**) were performed. *P < 0.05.

In this study, we also explored the effects of HDACi on YY1 expression. We measured YY1 expression in SMMC-7721 cells with SAHA and TSA treatments. QRT-PCR and western blot analysis showed that either SAHA or TSA could decrease the mRNA and protein levels of HDAC1 and YY1 (Figure 6A–6D, Supplementary Figure 3). Interestingly, overexpression of HDAC1 also up-regulated the mRNA and protein expression levels of YY1 in HEPG2 cells (Figure 6E and 6F, Supplementary Figure 4). These data suggested that there was a reciprocal regulation between YY1 and HDAC1.

## DISCUSSION

Long-term survival of HCC patients is still very low partly because of the high rate of intrahepatic invasion and metastasis. Moreover, HCC is usually known as a chemotherapeutic resistant tumor and patients with HCC usually exhibited poor tolerance to conventional chemotherapy due to the tumor heterogeneity and liver dysfunction [17]. In view of the facts above, new biomarkers for HCC diagnosis and treatment are urgently needed. HDACi has been used to treat cancers and exerted effective anti-tumor effects by modulating multiple signaling pathways [18]. HDACi was also reported to be a potential anti-HCC drug [19]. Although HDACis are currently being tested in diverse clinical trials for cancers, mechanisms underlining the efficacy of HDACi and predictive biomarkers for HDACi are still needed to be fully identified. In this study, we found that the level of transcription factor YY1 was associated with the sensitivity of HCC cells to HDACi. Further analysis showed that YY1 expression was positively correlated with HDAC1 in HCC cell lines and tumor tissues. These data suggested that YY1 would be a potential biomarker for HCC treatment with HDACi.

HDACi could induce cell cycle arrest, antiproliferation and apoptosis of HCC cells. In this study, we found that YY1 could attenuate the inhibitory effects of HDACi on HCC tumorigenesis. Considering the fact that YY1 was up-regulated in HCC tissues compared with ANLTs, we supposed that YY1 might act as an oncogene in HCC progression. Actually Tsang et al. have revealed that YY1 could promote proliferation and inhibit apoptosis of HCC cells [16]. Our results also indicated that HDACi decreased the expression of YY1 and YY1 would be a potential treatment target for HCC patients.

YY1 binds to DNA strands by a sequence-specific manner and plays important roles in development and differentiation [20]. YY1 regulates a variety of cancer-related genes including c-Myc, c-Fos and p53 [21–23]. C-terminal region of YY1 protein contains four C2H2 zinc fingers and is identified as one of the two repression domains of YY1, while the other repression domain located in the middle domain of YY1 protein [24]. The N-terminal domain of YY1 contributed to the activation of downstream genes [25]. Some reports have indicated that YY1 was capable of both activating and repressing gene transcription. For instance, YY1 could bind to the promoter of FEN1 and suppress its expression [12]. Another study showed that YY1 protein interacted with sequences of GR promoter and up-regulated its expression [26]. In this study, our experiments showed that YY1 could bind to HDAC1 promoter and increase its mRNA and protein levels. Until now, the specific mechanism underlying the bi-functional roles of YY1 in gene transcriptional regulation has not been fully determined.

HDACs are thought to bind to and deacetylate histones, and usually inhibit gene transcription by regulating chromosome condensation [27]. HDACs could not bind to DNA sequences directly, but they could interact with transcription factors which bind to the promoters of target genes. Interestingly, it was reported that YY1 could recruit HDACs on the promoter of target genes and regulate gene transcription [21]. On the other hand, Yao et al. reported that YY1 protein itself was deacetylated by HDACs in its acetylated central region and the acetylation of the central region was necessary for the full transcription repressor activity of YY1 [28]. These suggested that there was a complex relationship between YY1 and HDACs. In this study, we found that YY1 could directly bind to the promoter of HDAC1 and promoted its expression, while overexpression of HDAC1 in turn up-regulated the mRNA and protein levels of YY1. However, the specific mechanism underlying the effects of HDAC1 on YY1 expression is still needed to be explored.

In conclusion, our studies showed for the first time that YY1 was a potential biomarker for the sensitivity of HCC cells to HDACi and attenuated the inhibitory effects of HDACi on HCC tumorigenesis *in vitro* and *in vivo*. We also indicated that YY1 was positively correlated with HDAC1 in HCC cell lines and tumor tissues, while there was a reciprocal regulation between YY1 and HDAC1. Our study indicated potential roles of YY1 and HDAC1 in the clinical diagnosis and treatment of HCC patients.

## MATERIALS AND METHODS

### Cell culture

In this study, eleven HCC cell lines were used. MHCC97L and MHCC97H cells were purchased from Cobioer Biosciences Company (Nanjing, China). SMMC-7721, HUH-7, BEL-7404, QSG-7701, PLC/PRF/5, QGY-7701, HEPG2, QGY-7703 and HCCLM3 cells were obtained from Cell Resource Center of Shanghai Institutes for Biological Sciences, Chinese Academy of Sciences (Shanghai, China). All cells were cultured in DMEM medium containing 10% fetal bovine serum (Gibco, Grand Island, NY, USA) in a humidified atmosphere of 5% CO2 at 37°C.

### Tissue samples

Tissue samples were collected from patients with HCC who underwent liver resection at Department of Hepatobiliary and Pancreatic Surgery, the Affiliated Hospital of Qingdao University from June 2014 to October 2016. The clinicopathological data of HCC patients were shown in Supplementary Table 1. This work was carried out in accordance with the Declaration of Helsinki and was also approved by the Ethics Committee of Affiliated Hospital of Qingdao University. All participants provided their informed consents to participate in this study.

### Cell proliferation assays

Cell proliferation and IC50 values were determined by CCK-8 (Dojindo, Kumamoto, Japan) assay. Briefly, cells were seeded into a 96-well plate and cultured at 37°C for 24 h. Then cells were treated with SAHA (Sellekchem, Houston, TX, USA), TSA (Beyotime, Shanghai, China) or DMSO (Sigma, St. Louis, MO, USA). At various time points, CCK-8 was added into each well of the plate. The plates were incubated at 37°C for 30 mins and the absorbance at 450 nm was measured.

### Plasmids and transfection

YY1 and HDAC1 expressing plasmids were obtained from Biogot Technology (Nanjing, China). YY1 shRNA plasmids were purchased from Genechem (Shanghai, China). The efficiencies of three YY1 shRNA plasmids were shown in Supplementary Figure 5 and YY1-shRNA-1 plasmid was used for subsequent study. The transfection of plasmids was conducted using Lipofectamine 3000 (Invitrogen, Carlsbad, CA, USA).

### Quantitative reverse transcription-PCR (QRT-PCR)

Total RNAs of cells or tissues were extracted using Trizol (Invitrogen) according to the instructions provided by the manufacturer. After reverse transcription with Primescript RT Master Mix (Takara, Otsu, Japan), cDNA was amplified using SYBR-Green Premix (Takara). The expressions of YY1 and HDAC1 were normalized to the expression of β-actin. The data were analyzed by delta Ct method. Primers used in this study were listed: β-actin: 5′-CATCCTCACCCTGAAGTACCC C-3′ and 5′-AGCCTGGATGCAACGTACATG-3′; HDAC1: 5′-ACCCGGAGGAAAGTCTGTTAC-3′ and 5′-GGTAGAGACCATAGTTGAGCAGC-3′; HDAC2: 5′-ATGGCGTACAGTCAAGGAGG-3′ and 5′-TG CGGATTCTATGAGGCTTC A-3′; HDAC3: 5′-CGCCTG GCATTGACCCATAG-3′ and 5′-CTCTTGGTGAAGC CTTGCATA-3′; YY1: 5′-ATACCTGGCATTGACCT-3′ and 5′-TGAGGGCA AGCTATTGT-3′.

### Luciferase assay

A fragment from HDAC1 gene promoter was inserted into PGL3-Basic luciferase reporter vector (Promega, Madison, WI, USA). Mutant reporter plasmids were prepared by Mutagenesis Kit (Stratagene, La Jolla, CA, USA). Luciferase activity assay was conducted using Dual Luciferase Assay System (Promega). pRL-TK plasmid (Promega) was used to normalize the transfection efficiency.

### Chromatin immunoprecipitation (ChIP)

ChIP assay was conducted using EZ ChIP kit (Millipore, Billerica, MA, USA) and YY1 antibody (ab12132, Abcam, Cambridge, MA, USA) according to the instruction of the manufacturer. The primer specific to the HDAC1 promoter was listed: 5′-GGCTGGGTTCTGTCACTTTGTTAC-3′ and 5′-CCG CCAATGTTA TTTCAGTTTTTCC-3′. Normal rabbit IgG (Santa Cruz Biotechnology, Santa Cruz, CA, USA) was used to control the nonspecific immunoprecipitation.

### Apoptosis assay

Apoptosis was determined using Annexin V-FITC/propidium iodide (PI) apoptosis detection kit (Beyotime). Briefly, the treated cells were washed twice with phosphate-buffered saline and suspended in binding buffer. Then Annexin V-FITC and PI solution were added to cell suspensions and incubated for 15 mins at room temperature protecting from light exposure. The proportion of apoptotic cells was analyzed by flow cytometric analysis.

### Immunohistochemistry

Immunohistochemistry using streptavidin peroxidase-conjugated method was performed on paraformaldehyde-fixed paraffin sections. KI67 (bs2130R, BIOSS, Beijing, China) and HDAC1 antibody (ab109411, Abcam) were used in immunohistochemistry. Phosphate-buffered saline was used as a negative control instead of the primary antibody.

### Western blot

Total protein was extracted in NP-40 lysis buffer (Boster, Wuhan, China), separated by sodium dodecyl sulfate-polyacrylamide gel electrophoresis and then transferred onto 0.45 μm PVDF membrane (Millipore). The following primary antibodies were used in the immunoblotting assays: antibodies for YY1 (ab12132, Abcam), HDAC1 (ab109411, Abcam), HDAC2 (ab32117, Abcam), HDAC3 (ab32369, Abcam) and GAPDH (AG019, Beyotime). The quantitative analysis for western blot was conducted by using Image-J Software (NIH, Bethesda, MD, USA).

### HCC xenograft mouse model

YY1-shRNA lentivirus and NC lentivirus were purchased from Genechem (Shanghai, China). After infection, HCCLM3 cells were injected subcutaneously to the posterior flank of the BALB/c nude mice obtained from the Shanghai Institute of Materia Medica (Shanghai, China). Intraperitoneal injection of 100 mg/kg SAHA or DMSO in mice was conducted every day. About two weeks later, the mice were sacrificed. The tumor size was measured and calculated as follows: tumor volume (mm^3^) = (length × width^2^)/2. All animal studies were conducted in the Animal Institute of Qingdao University according to the protocols approved by the Medical Experimental Animal Care Commission of Qingdao University.

### Statistical analysis

Statistical analysis was performed using the SPSS program (version 18.0; SPSS, Chicago, IL, USA) and GraphPad Prism7 software (GraphPad Software, Inc., La Jolla, CA, USA). Data were presented as mean ± S.D. Statistical significance was calculated by Student's *t-test* or one-way ANOVA. Pearson's analysis was used in correlation analysis. *P* < 0.05 was considered as statistically significant.

## SUPPLEMENTARY MATERIALS FIGURES AND TABLES



## Abbreviations

HCC: Hepatocellular carcinoma
HDACs: Histone deacetylases
HDACi: HDAC inhibitor
ROS: reactive oxygen species
YY1: Yin-Yang 1
wt: wide type
mut: mutant
PI: propidium iodide.
