# Supplementary material for: YY1 promotes HDAC1 expression and decreases sensitivity of hepatocellular carcinoma cells to HDAC inhibitor
Source: Oncotarget. 2017 Apr 18;8(25):40583–93. doi: 10.18632/oncotarget.17196 (PMC5522268; doi:10.18632/oncotarget.17196)
Supplement: Supplementary file 1 [file oncotarget-08-40583-s001.pdf]

YY1 promotes HDAC1 expression and decreases sensitivity of hepatocellular carcinoma cells to HDAC inhibitor

Supplementary Materials

Supplementary Table 1: The clinicopathologic features of patients with HCC in this study

Factors	Number of patients
Age (≤ 60/> 60 years old)	25/25
Gender (male/female)	32/18
HBs Ag (Positive/Negative)	14/36
Cirrhosis (Yes/No)	17/33
Serum AFP level (≤ 400/> 400 μg/L)	37/13
Microvascular invasion (Absent/Present)	33/17
Number of tumors (1/≥ 2)	45/5
Tumor Size (≤ 5/> 5 cm)	30/20
Liver-Capsule Invasion (Yes/No)	23/27
Histological Differentiation (High/Moderate/Low Differentiation)	11/24/15
TNM stage (I/II–III)	37/13
BCLC stage (0–A/B–C)	38/12

Abbreviations: AFP, α-fetoprotein; BCLC, Barcelona Clinic Liver Cancer; HCC, hepatocellular carcinoma.

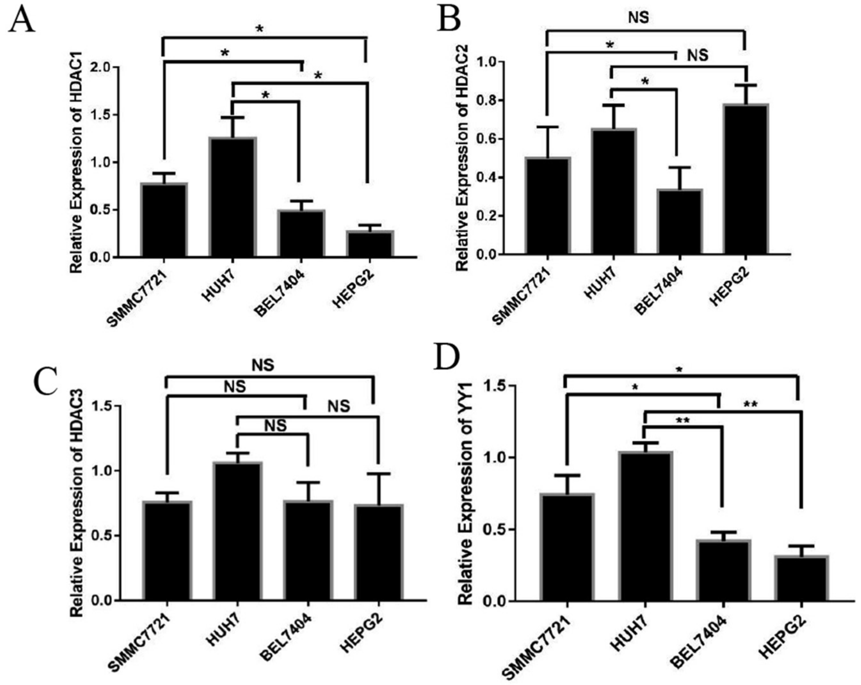

Supplementary Figure 1: Quantitative analysis of western blot for protein expressions of HDAC1, HDAC2, HDAC3 and YY1 in different HCC cell lines. Relative expression levels were expressed as the ratio of the density for HDAC1 (**A**), HDAC2 (**B**), HDAC3 (**C**) and YY1 (**D**) to the corresponding density for GAPDH. *$P < 0.05$; **$P < 0.01$; NS, not significant

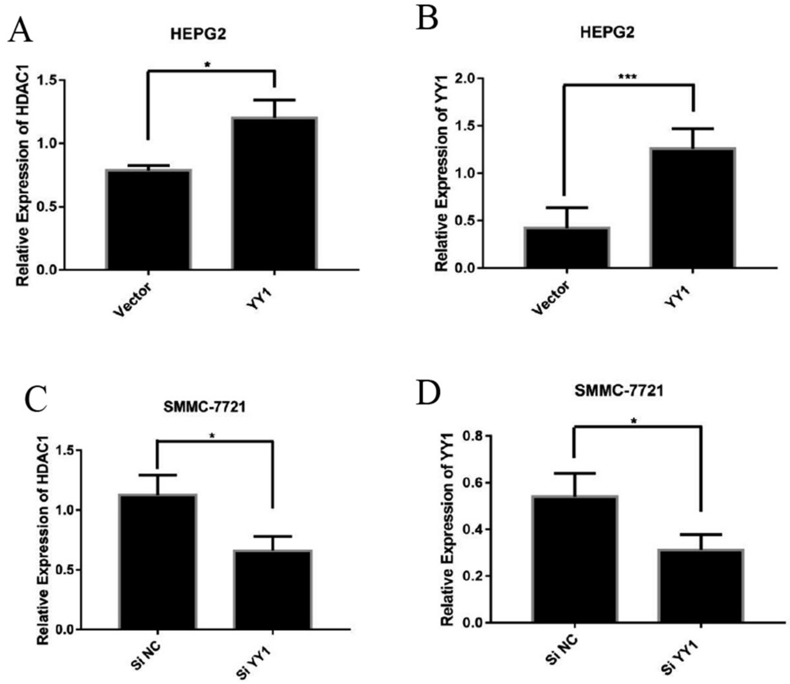

Supplementary Figure 2: Quantitative analysis of western blot for YY1 and HDAC1 expression in HEPG2 cells with YY1 overexpression (A–B) and SMMC-7721 cells with YY1 knockdown (C–D). Relative expression levels were expressed as the ratio of the density for YY1 or HDAC1 to the corresponding density for GAPDH. *$P < 0.05$; ***$P < 0.001$

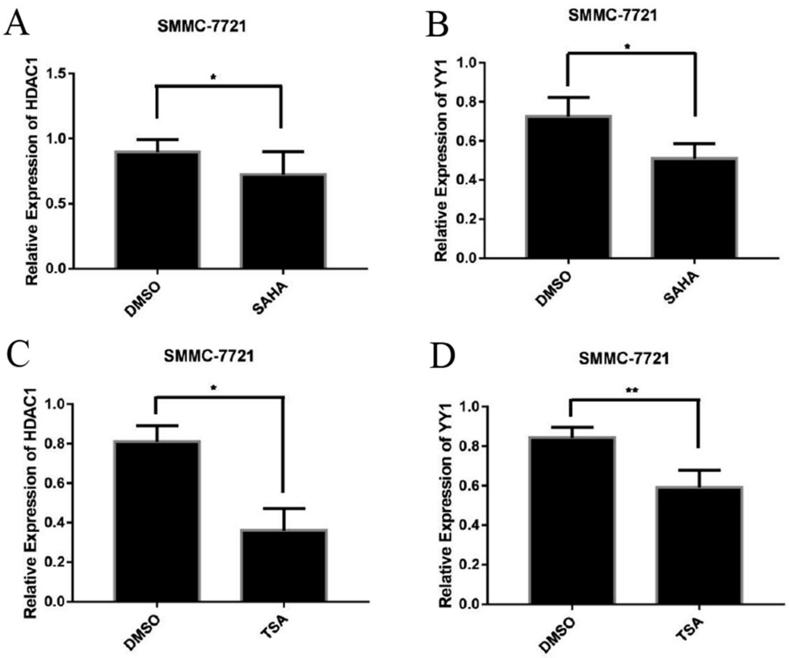

Supplementary Figure 3: Quantitative analysis of western blot for HDAC1 and YY1 expression in SMMC-7721 cells treated with 8μM SAHA (A–B) and 800nM TSA (C–D) for 48 h. Relative expression levels were expressed as the ratio of the density for YY1 or HDAC1 to the corresponding density for GAPDH. **P* < 0.05; ***P* < 0.01

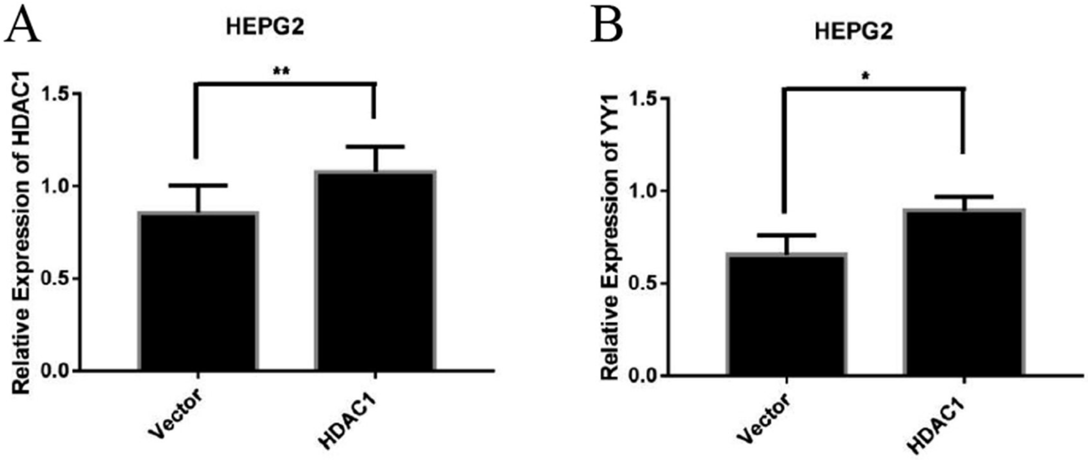

Supplementary Figure 4: Quantitative analysis of western blot for HDAC1 (A) and YY1 (B) expression in HEPG2 cells with HDAC1 overexpression. Relative expression levels were expressed as the ratio of the density for YY1 or HDAC1 to the corresponding density for GAPDH. **P* < 0.05; ***P* < 0.01

A

ShRNA(ID)	Target sequence
YY1-RNAi-1(22292)	ATGGTTGTAATAAGAAGTT
YY1-RNAi-2(22293)	CAAACAACTGGCAGAATTT
YY1-RNAi-3(22295)	CGACGACTACATTGAACAA

B

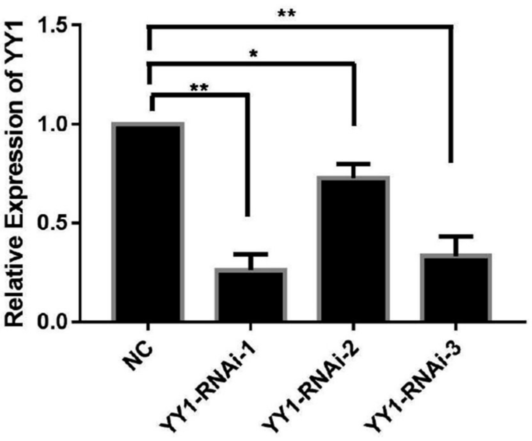

Supplementary Figure 5: Target sequences and inhibitory efficiency of three YY1-shRNA plasmids. (**A**) Target sequences of three YY1 shRNAs. (**B**) Relative mRNA expressions of YY1 in SMMC-7721 cells transfected with NC and three YY1 shRNA plasmids. The data were normalized to the expression level of YY1 in the cells transfected with NC. Results were represented as mean ± S.D. (n = 3). *$P < 0.05$; **$P < 0.01$
